# Damage effect of high-intensity focused ultrasound on breast cancer tissues and their vascularities

**DOI:** 10.1186/s12957-016-0908-3

**Published:** 2016-05-26

**Authors:** Liming Guan, Gang Xu

**Affiliations:** Department of Obstetrics and Gynaecology, Zhabei District Central Hospital, No. 619, Zhonghuaxin Road, Zhabei District, Shanghai, 200000 China; Department of Radiotherapy, Tumor Hospital, Peking University, No. 69, Wanfeng Road, Fengtai District, Beijing, 100000 China

**Keywords:** High-intensity focused ultrasound, Tumor vascularities, Breast cancer

## Abstract

**Background:**

High-intensity focused ultrasound (HIFU) is a noninvasive therapy that makes entire coagulative necrosis of a tumor in deep tissue through the intact skin. There are many reports about the HIFU’s efficacy in the treatment of patients with breast cancer, but randomized clinical trials are rare which emphasize on the systematic assessment of histological changes in the ablated tumor vascularities, while clinical trials utilizing bevacizumab and other anti-angiogenic drugs in breast cancer have not demonstrated overall survival benefit. The purpose of this study is to evaluate the damage effect of HIFU on breast cancer tissues and their vascularities.

**Methods:**

Randomized clinical trials and the modality of treat-and-resect protocols were adopted. The treated outcome of all patients was followed up in this study. The target lesions of 25 breast cancer patients treated by HIFU were observed after autopsy. One slide was used for hematoxylin-eosin (HE) staining, one slide was used for elastic fiber staining by Victoria blue and Ponceau’s histochemical staining, and one slide was used for vascular endothelial cell immunohistochemical staining with biotinylated-ulex europaeus agglutinin I (UEAI); all three slides were observed under an optical microscopic. One additional slide was systematically observed by electron microscopy.

**Results:**

The average follow-up time was 12 months; no local recurrence or a distant metastatic lesion was detected among treated patients. Histological examination of the HE slides indicated that HIFU caused coagulative necrosis in the tumor tissues and their vascularities: all feeder vessels less than 2 mm in diameter in the insonated tumor were occluded, the vascular elasticity provided by fibrin was lost, the cells were disordered and delaminated, and UEAI staining of the target lesions was negative. Immediately after HIFU irradiation, the tumor capillary ultrastructure was destroyed, the capillary endothelium was disintegrated, the peritubular cells were cavitated, and the plasma membrane was incomplete.

**Conclusions:**

HIFU ablation can destroy all proliferating tumor cells and their growing vascularities simultaneously; this may break interdependent vicious cycle of tumor angiogenesis and neoplastic cell growth that results in infinite proliferation. While it cannot cause tumor resistance to HIFU ablation, it may be a new anti-angiogenic strategy that needs further clinical observation and exploration. Furthermore, the treatment indications of HIFU ablation were reviewed and discussed in this manuscript.

## Background

### Evolution of breast cancer treatment

Breast cancer is the most frequently occurring malignant disease and leading cause of cancer-related death in women in the world, accounting for 7–10 % of the whole body all kinds of malignant tumors, tending to occur in 40- to 60-year-old women before and after menopause. Chinese females have a lower incidence of breast cancer compared with their counterparts in Western countries. However, the incidence of breast cancer has increased steadily at an amazing rate over the past two decades (from 29.9/100,000 in 1989–1993 to 50.1/100,000 in 2004–2008 in Chinese urban areas and from 6.5/100,000 to 17.3/100,000 in Chinese rural areas), making breast cancer the most common and the fifth most common cancer for Chinese urban and rural females, respectively [1].

Radical mastectomy, with or without excision of the pectoral muscle, has long been accepted as an appropriate therapy for breast cancer. This treatment was based on the theory that aggressive local therapy for control of breast cancer, chest wall, and regional lymph nodes would have a substantial benefit on survival [2]. In the 1970s, an increased understanding of the natural history of breast cancer resulted in the use of breast-conserving surgery, i.e., local excision for the treatment of breast cancer [3, 4]. Several randomized studies demonstrated similar survival rates for both treatment groups; breast-conserving surgery combined with radiotherapy became the standard treatment for patients with localized breast cancer. Beast cancer is a systemic disease, so hematogenous dissemination plays a very important role in the process [3]. Although breast-conserving surgery carries a relatively low morbidity rate, a variety of complications such as bleeding (2–10 %) and infections (1–20 %) can occur [4, 5].

Technological advances over the last decade have stimulated interest in even less invasive treatment of patients with localized breast cancer. One of the most attractive image-guided ablation therapies is high-intensity focused ultrasound (HIFU) ablation. HIFU ablation is a noninvasive procedure, i.e., requires no probe insertion and utilizes focussed ultrasound energy to coagulate tissue [6, 7]. HIFU ablation offers a promising method for noninvasive treatment of benign or malignant breast tumors. The breast is an organ with an excellent soft-tissue window that is required for the ultrasound beam to reach the target volume; furthermore, the breast can be easily immobilized [8, 9].

### Image-guided HIFU in the treatment of breast cancer

Ultrasound (US)- or magnetic resonance image (MRI)-guided HIFU therapy has been used to ablate localized solid tumors. Image-guided HIFU therapy may be the next important modality for real-time and effective guidance in breast cancers treated with HIFU. Several studies with different necrotic rates have shown HIFU to be effective and safe for breast cancer treatment. The complete necrosis rate observed is higher using ultrasound guided HIFU with fewer cases of skin burn [10].

Although the results of US-guided and MRI-guided HIFU ablation are promising, the data are too scant to justify a randomized controlled trial that compares HIFU ablation with breast-conserving surgery for treatment of localized breast cancer [10].

The next step towards clinical implementation of HIFU for breast tumor ablation would be a large prospective treat-and-resect study, namely, HIFU ablation followed by surgery, which allows histopathological tissue examination to evaluate the therapeutic effect of HIFU and provides data that can be used for technique standardization and guideline formulation.

### Anti-angiogenic therapy and breast cancer

Anti-angiogenic therapy pursues a strategy that is directed against the vascular constituent of the tumor stroma rather than the tumor cell itself. The tumor microenvironment as a primary target is less prone to resistance mechanisms due to genetic stability [11, 12].

Based on a review that revealed 75 clinical trials, completed or ongoing, utilizing anti-angiogenic drugs in early-stage breast cancer, among an increasing number of anti-angiogenic agents, bevacizumab remains the most established in the treatment of breast cancer. Clinical trials utilizing bevacizumab and other anti-angiogenic drugs in metastatic breast cancer have not demonstrated overall survival benefit. Most markers that have shown encouraging results in animal models have not correlated with therapeutic response or resistance in humans. Our current inability to identify which patients truly benefit from anti-angiogenic agents may be the real reason for the survival outcome of these trials [13, 14].

The reasons for a lack of overall survival advantage in all of these trials are unknown. Possible explanations include two general modes of resistance to angiogenesis inhibitors, in particular those targeting the vascular endothelial growth factor (VEGF) pathways: first, adaptive (evasive) resistance and second, intrinsic (preexisting) nonresponsiveness [15].

Since anti-angiogenic monotherapies may only act as cytostatic agents, making objective response measurements like tumor shrinkage, or simply inhibit further growth and spread. Then, further studies are needed to examine the effect of vessel “normalization,” whereby the tumor vessels become more organized and blood flow is improved and if improved tumor delivery of chemotherapeutics could be achieved [16].

Facing limitation of anti-angiogenic monotherapies (e.g., bevacizumab), other treatment methods such as HIFU may be recommended. HIFU caused coagulative necrosis in the tumor tissues, and their vascularities give another future prospect for anti-angiogenic therapy.

In this study, HIFU treatment would adopt “treat-and-resect” protocols to assess the therapeutic efficacy of US-guided HIFU ablation in patients with breast cancers and their vascularities and provides information for future breast cancer treatment and anti-angiogenic strategy. Twenty-five women with breast cancer confirmed by puncture pathology were enrolled in HIFU ablation followed by modified radical resection, 6 cases were in clinical stage I, 4 cases were in clinical stage IIA, and 15 cases were in clinical stage IIB. Another 25 women with breast cancer were performed modified radical resection as a control group.

## Methods

**Fig. 1:**
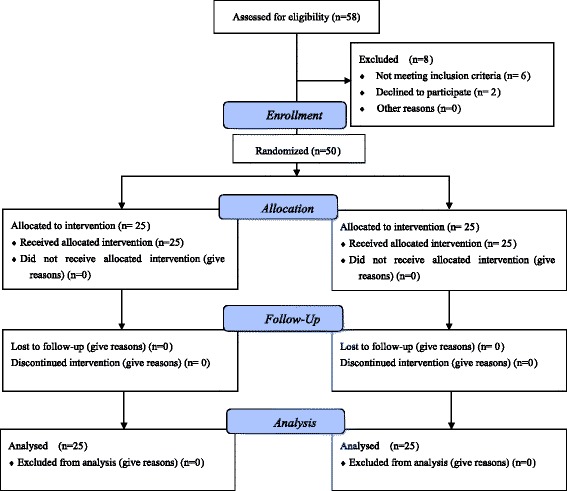
CONSORT flow diagram

From February 2014 to August 2014, 58 women with breast cancer patients were enrolled to Zhabei District Central Hospital, among whom 50 patients were included in our study. Fifty patients gave informed consent and were fully evaluated for eligibility to participate in the study. The inclusion and exclusion criteria were met by 50/58 (86.2 %) patients, and 8 (13.8 %) patients exited the study without treatment. Reasons for ineligibility included two patients refused treatment in Zhabei District Central Hospital; two patients with breast cancer accompanied by active myocardial disease and one with diabetes mellitus not controlled were not qualified for the operation; and one patient with breast tumors with undefined margins and two patients with breast multifocal tumors not suitable for HIFU treatment were excluded. Finally, fifty patients were admitted and randomized 1:1 to a treatment of modified radical mastectomy alone or modified radical mastectomy after HIFU ablation 1~2 weeks, see CONSORT flow diagram Fig. 1.

Our research was approved by the Ethics committee of Zhabei District Central Hospital (No.2014-85). All patients in this study were thoroughly informed of the benefits, curative effects, potential risks of the condition and management alternatives, treatment costs and uncertainties related to breast cancer-modified radical operation, or HIFU treatment followed by surgery. All patients gave written consent to participate in the study. There were no conflicts of interest.

The patients’ inclusion criteria were as follows: (1) pathology proven invasive breast cancer (T1-2, N0-2, M0); single palpable tumors less than 5 cm in diameter; the lesion boundaries visualized with color doppler ultrasound imaging, circumscribed at least more than 1.0 cm from the skin or rib cage, and more than 2 cm from nipple and (2) patients with breast cancer were without coagulation disorders and myocardial disease, diabetes mellitus not controlled, or other complicated diseases. Patients’ vital signs were stable when admitted to the hospital.

The diagnosis of breast cancer was based on breast puncture pathology. Sentinel lymph node (SLN) biopsy was performed at the same time.

After appropriate counseling, patients were referred to our breast cancer treatment center. Both patients and doctors were blinded to group allocation. Enrolled patients were then sequentially numbered. Patients labeled with odd numbers were given modified radical mastectomy alone, while patients labeled with even numbers received modified radical mastectomy after HIFU treatment.

Control group: twenty-five patients with breast cancer confirmed by puncture pathology were enrolled in this study. Modified radical mastectomy was performed for all patients without any other treatment before surgery operation. Patient age ranged from 25 to 65 years, and tumor diameter ranged from 2.3 to 4.5 cm. Eleven cases had ipsilateral lymph node metastasis. Seven cases were in clinical stage I, 5 cases were in clinical stage IIA, and 13 cases were in clinical stage IIB.

HIFU group: twenty-five patients with breast cancer confirmed by puncture pathology were enrolled in this study. Modified radical mastectomy was performed 1~2 weeks after HIFU extraneous ablation of breast cancer in situ. Patient age ranged from 22 to 63 years, and tumor diameter ranged from 2.1 to 4.8 cm. Twelve cases had ipsilateral lymph node metastasis. Six cases were in clinical stage I, 4 cases were in clinical stage IIA, and 15 cases were in clinical stage IIB.

Modified radical mastectomy consisted of excising the whole breast tissue off the chest wall including the axillary tail and complete axillary nodes dissection and connective tissue up to level 3 by generous retraction of the pectoralis minor muscle.

Patients’ demographic and tumor characteristics were recorded.

Two weeks follow-up after HIFU ablation was performed to assess the potential side effects of HIFU such as skin burns, local pain or discomfort, mammary edema, hemorrhage or infection, and fever. After surgery, all patients received relevant doses of local radiation therapy, conventional chemotherapy, and hormonal therapy, as part of their assistant treatment.

The study was primary planed to follow patients for every year for up to 5 years. The treated outcome of patients in both groups was followed up in this study. The rate of local recurrence and distant metastasis was detected in both groups.

**Fig. 2:**
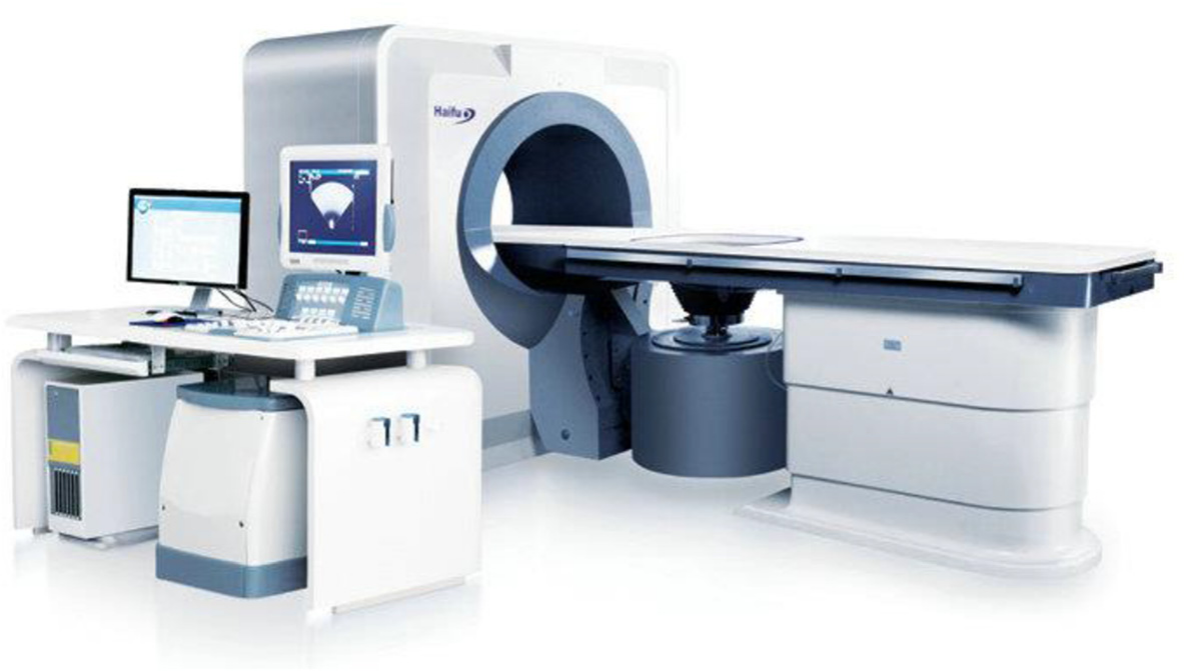
The high-intensity ultrasonic JC tumor treatment system

Treatment device and its components: the high-intensity ultrasonic JC tumor treatment system was designed and made by Haifu Medical Technology co., LTD, Chongqing, China (Fig. 2). The HIFU therapeutic system used for this breast cancer treatment principally consists of a diagnostic ultrasound device, units for computer automatic control, six-direction movement, a therapeutic planning system, an ultrasound generator, integrated ultrasound therapy transducers, and a degassed water circulation unit. The therapeutic ultrasound energy was produced by a 12-cm-diameter PZT-4 piezo-ceramic transducer (Beijing Cheng-Cheng Weiye Science and Technology Co., Ltd, Beijing, China). The main parameters of the breast ablation were configured as follows: 1.6 MHz, focal lengths 90 mm, focal zone (transverse × axial) 3.3 × 1.1 mm, and a diameter of the transducer surface of 200 mm. The sound intensity was 5000~20,000 W/cm^2^, a therapy power of 300–400 W.

An AU3 ultrasound imaging device (Esaote, Genoa, Italy) was used as the real-time imaging unit of the HIFU therapeutic system. This imaging probe (3.5–5.0 MHz) was placed at the center of the HIFU transducer for real-time guidance during the HIFU treatment procedure. The ultrasound beams of the therapeutic transducer and the imaging probe completely overlapped so that the longitudinal axis of the HIFU beam was in the two-dimensional ultrasound imaging plane. The integrated transducer was attached to electric motors and could be moved smoothly in six directions with millimetric precision. Through computer control, the imaging transducer was placed either against the skin or at a particular distance from the skin in the water for pretreatment imaging.

HIFU treatment process: after initiating general anesthesia with the patient fixed in a movable bed, the patient was placed prone position at the surface of the acoustic coupling agent (degassed water) treatment bed. The ultrasonic wave went through the skin and subcutaneous tissue and converged at the breast tumor tissue. First, contrast ultrasonography was used for monitoring, with the central focus at the target area. Then, the HIFU treatment system continuously ultrasonically scanned the target region, which included tumor and 2 cm of breast tissue surrounding the tumor. Sonograms obtained immediately after the treatment were compared to those obtained before the treatment in terms of echo intensity changes to determine the scope of the tumor tissue damage after HIFU treatment. Ultrasonographic changes after HIFU treatment that indicated that the treatment was effective included a strong echo area and the disappearance of blood flow.

By scanning the HIFU beam in successive sweeps from the deep to the shallow regions of the tumor, the targeted regions on each slice were completely ablated. The 150 W of test power was first used and then gradually increased up to 300–400 W. One sonication lasted for 5 s. With the help of provisional therapeutic parameters based on the depth and vascular supply of the target region, the process was repeated slice by slice to achieve ablation of all planned slices. Grayscale changes (echogenicity) on the diagnostic images within the focus after each HIFU exposure were analyzed to identify and monitor the extent of treatment.

In this study, the scanning speed ranged 1–3 mm/s, and the track length was 20 mm. HIFU ablation time ranged from 40 min to 2.2 h (median, 1.1 h).

### Breast cancer pathological observation

All removed specimens including breast tumor and normal breast tissue were evaluated by gross and histological observations. They were serially sectioned at approximately 5-mm intervals, and the slice thickness was measured. Gross observations were recorded in a protocol containing the overall appearance, size, and shape.

The tumor tissues in the HIFU treatment group and the control group were fixed in 10 % neutral formaldehyde, conventionally paraffin embedded, sectioned, and placed on slides. Then, one slide was stained with hematoxylin and eosin (HE), one slide was stained with Victoria blue to observe elastic fibers and Ponceau’s histochemical stain, and one slide was biotin-labeled and stained with ulex europaeus agglutinin (UEA) I. All slides were observed under an optical microscope.

### Elastic fiber staining

The reagent preparation methods for Victoria blue and Ponceau’s histochemical staining were described previously references [17].

Elastic fiber staining method: the biopsy was dewaxed in water. A section was washed in 70 % ethanol for 2 min and then incubated with the Victoria blue solution for 0.5~2 h. The Spring red S dropped slice 2 min, flushed extra dyeing liquid two times with anhydrous ethanol directly. The slice was dried in the air, xylene transparent, and neutral gum cementing.

For the slide that was dyed for elastic fiber damage, a microscopic micrometer was used to measure the tumor vascular caliber.

The microscopic micrometer had two parts that were used cooperatively: eyepiece micrometer and stage graticules. For the microscopic measurements, first, the stage graticules were used to measure the length of each grid in the ocular field; then, the eyepiece micrometer was used to measure blood vessel diameter. At ×100 and ×400 magnifications, each eyepiece micrometer represented 4.8 and 1.2 μm, respectively. We first looked for blood vessels at ×40 magnification and then looked for vascular damage at ×400 magnification. For each specimen, five horizons were counted, and the average vascular caliber of HIFU damage was calculated.

Blood vessels that had a thick muscle layer or a vascular diameter greater than eight red blood cells were examined by HE staining. Elastic fibers were stained and observed separately. The tumor blood capillaries, venules, and arterioles were examined at ×400 magnification under a light microscope. The five highest blood vessels (including capillaries, venules, and arterioles counts summing up) per tumor region were averaged and used for as the statistical analysis.

### Tumor-derived vascular endothelial UEAI staining

The avidin-biotin peroxidase complex (ABC) method was adopted. Biotin-labeled UEAI (Beijing Zhongshan Biotechnology Co., LTD) was used at a working concentration of 20 μg/ml. UEA can be combined with diaminobenzidine (DAB) coloration of endothelial cells.

UEA staining and microvessel density count: isolated endothelial cells and cell clusters that were positively stained with UEA with or without visible lumina were counted as separate microvessels. The tumor microvessel region was examined at ×100 magnification under a light microscope. Microvessel density was examined at ×400 magnification under a light microscope. The five highest microvessel density counts per tumor region (called “hot spots”) were averaged and used for the statistical analysis.

### Evaluation of VEGF expression

The VEGF staining by immunohistochemistry was manipulated as anti-rabbit vascular endothelial cell growth factor (Shanghai Yinggong enzyme-linked detection reagent co., LTD) in dilution 1:100 staining. The VEGF expression was evaluated semiquantitatively by calculating the sum of the scores from dyeing range and intensity. The scores of dyeing range followed the criteria: 1+, 0~10 % dyeing cells; 2+, 10~50 % dyeing cells; 3+, 50~75 % dyeing cells; and 4+, 75~ 100 % dyeing cells. The scores of dyeing intensity were calculated as follows: 1+, mild (the dyeing intensity in the cells was weak and even defect); 2+, moderate (the amount of cells evenly stained bronzing particles was less than that of 10 % total cells in the area); and 3+, serious (the amount of cells evenly stained bronzing particles was more than that of 10 % total cells in the area).

### Ultrastructural observation

Specimens were cut into 1-mm^3^ pieces. Then, half of the piece was prepared by the CQR-1 type ultra-microtome method, and ultrathin sections were prepared after positioning. The sections were stained with uranyl acetate and lead nitrate and observed and photographed by H-600 transmission electron microscopy.

### Statistical analysis

The data in the present study were showed as mean ± SD. Student *t* test and correlation analysis were used to analyze the variance in different groups; SPSS 19.0 software (SPSS, Inc.) was used for the statistical analyses of the data. The accepted level of significance was set at *p* < 0.05.

## Results

### Patients’ demographic characteristics

Patient demographic characteristics of both groups are shown in Table 1, and the majority of patients were postmenopausal women from Shanghai’s urban residents with no prior history of cancer and no family history of breast cancer.

**Table 1:** Patient demographic characteristics

	Control group (*n* = 25)	HIFU group (*n* = 25)	*p* value
Age: mean (range)	45 years (25~65)	48 years (22~63)	*p* = NS
Urban/rural resident			
Urban resident	17 (68.0 %)	16 (64.0 %)	*p* = NS
Rural resident	8 (32.0 %)	9 (36.0 %)	
Menopausal status: *n* (%)			
Premenopausal	5 (20.0 %)	4 (16.0 %)	*p* = NS
Peri-menopausal	4 (16.0 %)	5 (20.0 %)	
Postmenopausal	16 (64.0 %)	16 (64.0 %)	
Familial history of breast cancer: *n* (%)			
No family history	16 (64 %)	17 (68.0 %)	*p* = NS
First degree relative with breast cancer	6 (24 %)	5 (20.0 %)	
Not reported	3 (12 %)	3 (12 %)	
Prior history of cancer: *n* (%)			
Yes	2 (8.0 %)	3 (12.0 %)	*p* = NS
No	17 (68.0 %)	17 (68.0 %)	
Not reported	6 (24.0 %)	5 (20.0 %)	

*NS* nonsignificant

Both groups of patients were not significantly different in demographic characteristics.

### Tumor characteristics

Tumor characteristics of both groups are shown in Table 2. The pathological TNM staging in both groups was as described above.

**Table 2:** Breast cancer characteristics

	Control group (*n* = 25)	HIFU group (*n* = 25)	*p* value
Tumor size: mean (range)	2.3~4.5 cm	2.1~4.8 cm	*p* = NS
Breast cancer TNM staging: *n* (%)			
Stage I	7 (28.0 %)	6 (24.0 %)	*p* = NS
Stage IIa	5 (20.0 %)	4 (16.0 %)	
Stage IIb	13 (52.0 %)	15 (60.0 %)	
Not reported	0 (0.0 %)	0 (0.0 %)	
Histopathologic grade			
G1 well differentiated	12 (48.0 %)	10 (40.0 %)	*p* = NS
G2 moderately differentiated	9 (36.0 %)	12 (48.0)%	
G3 poorly differentiated	4 (16.0 %)	3 (12.0 %)	
Receptor status			
ER+/PR+	14 (56.0 %)	13 (52.0 %)	*p* = NS
ER+/PR−	2 (8.0 %)	4 (16.0 %)	
ER−/PR−	8 (32.0 %)	7 (28.0 %)	
Histological subtype			
Infiltrating ductal carcinoma	14 (56.0 %)	13 (52.0 %)	*p* = NS
Infiltrating lobular carcinoma	5 (20.0 %)	7 (28.0 %)	
Other subtype	6 (24.0 %)	5 (20.0 %)	
Ipsilateral lymph node metastasis			
Sentinel lymph node biopsy	10 (40.0 %)	10 (40 %)	*p* = NS
Postoperative permanent pathology	11 (44.0 %)	12 (48 %)	
Not reported	0 (0.0 %)	0 (0 %)	
Lesion location: side			
Left side	12 (48.0 %)	14 (56.0 %)	*p* = NS
Right side	13 (52.0 %)	11 (44.0 %)	
Lesion location: vertical			
Upper	7 (28.0 %)	9 (36.0 %)	*p* = NS
Lower	11 (44.0 %)	10 (40.0 %)	
Midline	7 (28.0 %)	5 (20.0 %)	
Lesion location: horizontal			
Upper	7 (28.0 %)	9 (36.0 %)	*p* = NS
Lower	11 (44.0 %)	10 (40.0 %)	
Midline	7 (28.0 %)	5 (20.0 %)	

*ER+* estrogen receptor positive, *ER−* estrogen receptor negative, *PgR+* progesterone receptor positive, *PgR−* progesterone receptor negative, *cm* centimeters, *NS* nonsignificant

Axillary node metastasis was screening by SLN biopsy before operation first, and then, it was proved by postoperative pathology. One false-negative case was found by postoperative pathology in control group (4 %), two false-negative cases were found by postoperative pathology in HIFU group (8 %), so SLN biopsy false-negative rate was about 4–8 % of the axillary node-positive patients.

Invasive ductal carcinoma was the commonest histological subtype in 27 (54.0 %) patients in both groups. Immunohistochemistry results were available for the tumors of 50 patients in both groups. Of these, 33 (66.0 %) were estrogen receptor (ER) positive and 25 (50.0 %) were progesterone receptor (PR) positive. It was not the main subject of this study, so detailed information was omitted.

Both groups of patients were not significantly different in tumor pathologic characteristics.

### Tumor vascular characteristics and the effect of HIFU treatment

#### HE staining

**Fig. 3:**
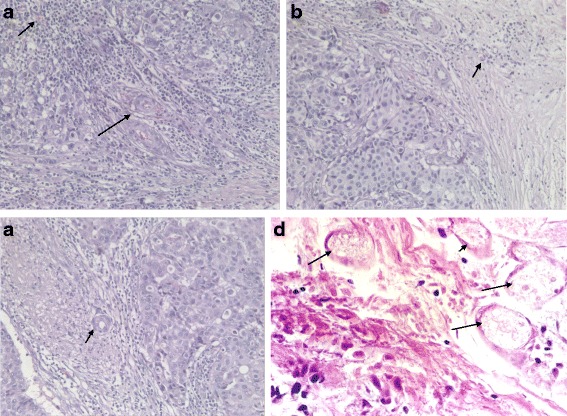
**a** Control group: blood capillary and venule inside the breast cancer nest were integrated (marked with *arrows*, 400 times HPF). **b** Blood capillary peripheral to the breast cancer nest were integrated (marked with *arrows*, 400 times HPF). **c** Arteriole peripheral to the breast cancer nest were integrated (marked with *arrows*, HE staining, 400 times HPF). **d** After HIFU treatment, cancer cell nucleus pyknosis, diffusion cellular arrangement, and vascular thrombosis are observed (marked with *arrows*, HE staining, 400 times HPF)

Control group: blood vessels (including tumor blood capillaries, venules, and arterioles) inside and peripheral to the breast cancer nest were integrated (Fig. 3a–c).

Control group including 25 cases of patients, and the average tumor capillary, venule, and arteriole amounts summing up under a light microscope at ×400 magnification, was 17.20 ± 3.50/vessels/high-power field (HPF), see Fig. 5c.

HIFU group: all HIFU-ablated tumors in the 25 patients were verified by gross observation after excision of the diseased breast. The ablated area without bleeding and injury was found; this proved that HIFU ablation was safe. Further macroscopic observation revealed that HIFU ablation caused coagulative necrosis of the targeted tissue thoroughly, which included the tumor and average margin of 1.92 ± 0.35 cm (between 1.8 and 2.3 cm) of an apparently normal breast tissue surrounding the tumor. Thermal effect of HIFU made ablated tissue touch stiffer, and there was an inflammatory reaction band at the margin between the treated and untreated regions.

Breast cancer specimens were divided at horizontal axis, and postoperative examination was obtained with slicing randomly and double staining with both hematoxylin-eosin and UEAI immunohistochemistry (see next).

Histological observation showed uniform coagulative necrosis, including the tumor and common breast tissue within the target region. It is not found residual viable tumor cells at the center of breast cancer tissues and at the margin of breast cancer tissues after HIFU treatment.

Immediately after HIFU treatment, cell damage was mainly characterized by cell pyknosis, nuclear pyknosis with heavy staining, significantly widened cell gaps, and intact cellular contours. Vascular structure in the HIFU group was thoroughly destroyed. Appearances were blurry cellular margins, endothelial disruption, nuclear vanish, and damage of tunica media, demonstrative of coagulative necrosis. The damaged vessels included capillaries, venules, and arterioles. Scattered intravascular thrombi were often observed in the destroyed vessels and initiation of dissolution of the tumor; irreversible cell necrosis was observed at 24 h after HIFU treatment (Fig. 3d, Fig. 5c).

#### Elastic fiber staining vascular

**Fig. 4:**
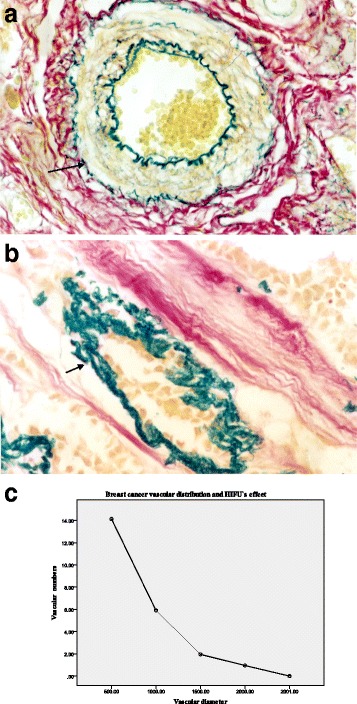
**a** Control group: integrity of internal and external elastic plates of blood vessels (elastic fiber marked with *arrows*, double histochemical staining of elastic fibers and collagen fibers in blood vessels: elastic fibers are *blue*, collagen fibers are *red*, background is *pale yellow*, tube diameter is 100 μm, ×400 HPF). **b** After HIFU treatment, elastic fiber disintegration is observed in blood vessels (elastic fiber marked with *arrows*, double histochemical staining of elastic fibers and collagen fibers in blood vessels: elastic fibers are *blue*, collagen fibers are *red*, background is *pale yellow*, tube diameter is 100 μm, ×400 HPF). **c **The diameters of the tumor blood vessels were all within 2000 μm in both groups, the majority of vascular diameter within 500 μm; smaller vascular diameter was positively correlated with more obvious damage by HIFU

For blood vessel histochemical staining, elastic fibers stained blue and collagen fibers stained red with a pale yellow background. For the control group, the internal and external elastic plates of the breast cancer tumor blood vessels were intact (Fig. 4a). For the HIFU treatment group, the elastic fibers in the blood vessels appeared to be disintegrated with an uneven distribution, fracturing, and layering (Fig. 4b). The diameters of the tumor blood vessels were all within 2 mm in both groups. The amount and distribution of vascular damage after HIFU treatment are shown in Table 3, Fig. 4c: smaller vascular diameter was positively correlated with more obvious damage by HIFU. In the control group, neither the tumors nor their blood vessels were damage, and the elastic fibers did not appear to be destroyed.

**Table 3:** HIFU’s vascular damage amount and distribution of vascular diameter

Vascular diameter	Control group (*n* = 25)	HIFU group (*n* = 25)	*t* value	*p* value
≤500 μm	14.13 ± 2.71	0	14.71	0.000
501~1000 μm	5.91 ± 1.5	0	10.24	0.000
1001~1500 μm	1.95 ± 1.43	0	6.41	0.000
1501~2000 μm	0.95 ± 0.84	0	5.306	0.000
>2001 μm	0	0	0	0

#### UEAI detection in endothelial cells

**Fig. 5:**
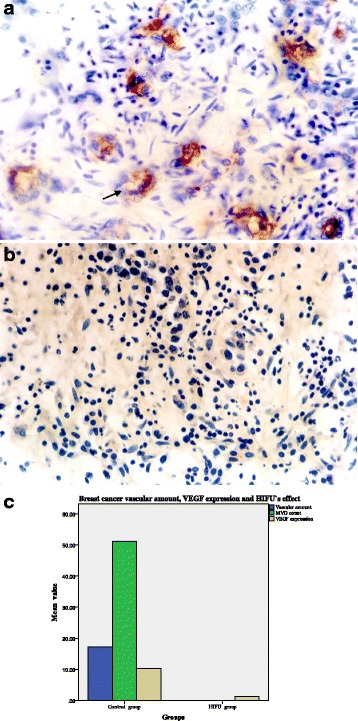
**a** The control group was positive for endothelial cell UEAI expression (endothelial cell marked with *arrows*, ×400 HPF). **b** The HIFU group was negative for endothelial cell UEAI expression (×400 HPF). **c** The average tumor capillaries, venules, and arterioles amount together 17.20 ± 3.50/vessels/HPF for the control group; vascular structure was destroyed for the HIFU group, with a vascular amount 0; UEAI staining was positive in control group, microvessel density count (MVD) was 51.18 ± 9.56/vessels/HPF, and UEAI staining was negative for the HIFU group, with a MVD of 0; the expression of vascular endothelial growth factor (VEGF) expression for the control group and HIFU group were 10.23 ± 2.20 and 1.31 ± 0.65, respectively; VEGF expression for the HIFU group remarkably decreased

Control group: microvessel UEAI staining was positive, with an uneven distribution in the tumor tissue, a high density of blood vessels, called “hot spots,” a lumen, or a single endothelial cell or cluster of endothelial cells (Fig. 5a). The control group included 25 cases, and the average microvessel density count under a light microscope at ×400 magnification was 51.18 ± 9.56/vessels/HPF.

For the HIFU group, endothelial UEAI staining was negative (Fig. 5b), with a vascular density count of 0. The *t* test values of the comparison of the two groups were *t* = 25.085 and *p* = 0.000, indicating a statistically significant difference (Fig. 5c).

As shown in Fig. 5c, the expression of vascular endothelial growth factor (VEGF) expression in control group and HIFU group was 10.23 ± 2.20 and 1.31 ± 0.65, respectively; there was significant difference between control group and HIFU group; and VEGF expression level in HIFU group remarkably decreased (*t* = 11.14, *p* < 0.000).

#### Electron microscopy observation

**Fig. 6:**
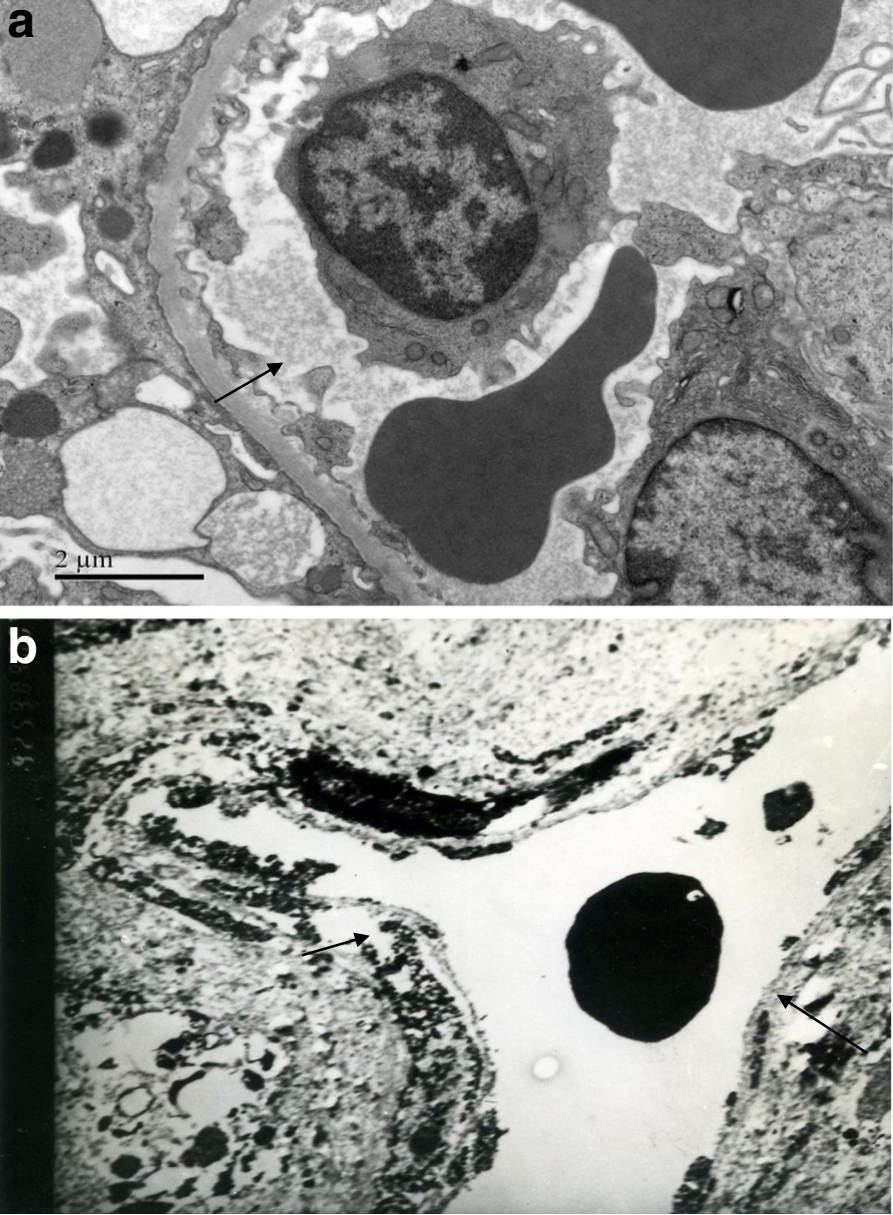
**a** Control group: vascular endothelial cell organelles of tumor were integrity (endothelial cell marked with *arrow*, transmission electron microscope images, uranyl acetate, and lead nitrate staining, ×15,000). **b** After HIFU treatment, the following were observed: capillary endothelial disintegration, peritubular cell cavitation, and incomplete plasma membrane (endothelial cell marked with *arrow*, transmission electron microscope images, uranyl acetate, and lead nitrate staining, ×6000)

Control group: vascular endothelial cell organelles of tumor were integrity (Fig. 6a). Immediately after HIFU irradiation, the tumor capillary ultrastructure was destroyed, the capillary endothelial appeared to be disintegrated, the peritubular cells were cavitated, and the plasma membrane was incomplete (Fig. 6b).

### Postoperative follow-up

Patients in HIFU group were first received HIFU treatment, no severe complications were found in 25 patients after HIFU treatment. Edema appeared in the mammary tissue circumjacent the ablated tumor. In all patients, the ablated region included the tumor and about 2.0-cm margin of normal breast tissue around the tumor. The edema gradually disappeared about 7 days later. No infection, even hemorrhage occurred at the treatment site. Eleven cases (44.0 %) were complaint of pain, tenderness, or discomfort and 3 cases (12 %) had mild fever over 38 °C within 48 h after operation, but these all were transitory. This lasted 1~3 days, and the pain and fever were successfully controlled by tramadol intramuscular injection or indomethacin suppositories.

Patients in both groups were received modified radical mastectomy.

Postoperative complications within 30 days of surgery occurred in six (12.0 %). Surgical wound infection occurred in two (4.0 %) patients.

The surgical drains were removed with a mean of 10 days (8~14 days) after operation when the drainage liquid was clear and less than 30 ml in the preceding 24-h period. None of the patients was discharged from hospital with the drain. Duration of hospital stay from admission for mastectomy to discharge postoperatively was with a mean (SD) of 12 days (9~15 days) for patients in control group, while duration of hospital stay prolonged 7~14 days for patients in HIFU group.

The study was first planed to follow patients for every year for up to 5 years. Twelve months of follow-up data have been collected for 50/50 (100 %) patients. No patients were lost to follow-up in both groups. Fifty patients agreed the follow-up period of the study. One-year data are available in all of 50 patients, with a median duration of follow-up of 368 days. There were no episodes of recurrence at the most recent follow-up visit in these 50 patients in both groups (Fig. 1).

The average follow-up time was 12 months in this study, the rate of local recurrence and distant metastasis were detected in both groups, and a local recurrence or a distant metastatic lesion was not detected in both groups. Because the follow-up period was short, the overall survival of HIFU treatment investigation in this study will be continued.

## Discussion

### Anti-angiogenic strategy and HIFU ablation

Anti-angiogenic method is a strategy that is directed against the vascular constituent of the tumor stroma rather than the tumor cell itself. The tumor microenvironment as a primary target is thought less likely growing resist to the anti-angiogenic agents due to genetic stability [18].

But the whole course of tumor vasculogenesis is far from complex than first expected. Experimental and clinical observation showed that blocked tumor vasculogenesis can inhibit tumor growth in vivo, while cancer cells can escape anti-angiogenic therapy and develop immunity to sole treatment. So clinical trials utilizing bevacizumab and other anti-angiogenic drugs in breast cancer have not demonstrated overall survival benefit at present time [19].

Stromal components in tumor microenvironment contribute centrally to tumor progression and metastasis. Reciprocal interactions occur between neoplastic cells and stromal components leading to coevolution. Tumor angiogenesis and tumor regeneration are interdependent and form a vicious aggravating cycle that results in infinite proliferation. Only killing all proliferating tumor cells and their feeding blood vessels simultaneously can eradicate a tumor [15, 16, 18, 19].

We performed randomized clinical trials and systematically assessed histological changes of HIFU ablation breast cancer and their vascularities. It is demonstrated that HIFU treatment can irreversibly stop tumor growth and the resulting hyperplasia vascular necrosis while no severe complications were found in all 25 cases of patients after HIFU sonication. Mild complications include edema, mild fever, complaint of pain, these all were transitory, which were successfully controlled by symptomatic treatment. No infection and no hemorrhage occurred at the treatment site of breasts.

The key to HIFU treatment of tumors is to first form a single coagulation necrosis zone within the organized mass, called the biological focal region (BFR). A conformal scanning “BFR” probe has been used to achieve complete resection of tumors in vitro, so the “BFR” is the basic unit for HIFU treatment of tumors [20].

HIFU lesion area size, focal region sound intensity, irradiation time, and the amount of irradiation are all positively correlated. Small (<1 cm^3^) tumors can be completely eradicated by a single pulse of irradiation. However, a single HIFU irradiation treatment cannot completely destroy larger tumors, which require multiple pulses of HIFU irradiation at multiple damage points closely arranged in a matrix to form a larger damage area that completely encompasses the tumor [21].

The application of HIFU treatment for breast cancer is performed under ultrasound real-time monitoring. The tumor is continuously irradiated by scanning the tumor, and each irradiation area is closely arranged to form a matrix resulting in damage to the adjacent area overlapping the tumor area to an extent that avoids residual damage to the area as much as possible. Irradiation continues until the tumor is completely contained within the HIFU irradiation range, as indicated by the observation of ultrasonographic changes to the tumor and the pathological changes from before to after irradiation. These findings are used to determine the efficacy of the treatment. After HIFU treatment, the ultrasonogram showed a strong echo in the entire area of the tumor and the absence of a blood supply to the tumor [8–10, 20, 21].

HIFU damage to blood vessels is related to their vascular diameter, blood flow velocity, and ultrasonic characteristics (frequency, intensity, and irradiation time). The damage is caused by thermal and cavitation effects as mentioned above [22].

Yang et al. used a rat liver animal model to examine HIFU treatment with microvascular imaging technology and found that tumor microvessels of less than 0.2 mm in diameter were immediately destroyed by HIFU (4 MHz, 550 W/cm^2^, 4 s). He stated that HIFU can only cause vascular damage to blood vessels of less than 0.2 mm in the liver. Delayed damage between HIFU irradiations in the necrotic areas may be due to secondary microcirculation damage in the irradiated area. In the process of primary and secondary liver cancer treatment, blood vessels are very important. Reducing or eliminating the tumor blood supply causes tumor necrosis [23].

HIFU treatment can damage nearby great vessels, which is always a cause of concern. Yang et al. used an in vivo rabbit abdominal aorta and inferior vena cava model to perform a HIFU slow treatment experiment (4 MHZ, 1500 W/cm^2^, 5 s), and the results of that experiment showed that HIFU did not cause injury to nearby blood vessels because the large blood vessels, such as the abdominal aorta, have wider diameters with faster blood flow (blood flow has a cooling effect). The experimental results showed that HIFU was relatively safe for large blood vessels [23].

Our HIFU treatment system achieved accurate ultrasonic positioning. In the 25 breast cancer patients treated by HIFU, no vascular rupture bleeding during the intraoperative or postoperative period occurred in one case. Our findings confirm that HIFU treatment is safe for nearby great vessels and that HIFU with real-time monitoring guided by ultrasound can effectively kill breast tumors and their nutrient vessels without harming the surrounding tissues. Additionally, this technique is highly controllable. Therefore, HIFU has good potential for clinical application as a new technology for the localized treatment of breast cancer.

Because the main mechanism of HIFU treatment is the exposure of tumor cells to high temperatures and the promotion of blood protein coagulation leading to tumor tissue coagulation necrosis, all tumor types exhibit highly similar basic pathological changes after HIFU treatment. Additionally, smaller tumor vessel diameter is positively correlated with better destruction of the tumor by HIFU. With a sound intensity probe set at 5000~20,000 W/cm^2^, HIFU can completely destroy all vessels in the target tumor and tumor parenchyma tissue that have a diameter of less than 2 mm.

Our HE staining results showed that HIFU treatment immediately caused cell damage, mainly characterized by cell pyknosis, nuclear pyknosis with heavy staining, significantly widened cell gaps, intact cell contours, and tumor vascular thrombosis. Additionally, tumor dissolution through necrosis was found at 24 h after HIFU treatment.

All tumor blood vessels had diameters of 2 mm or less in both groups. Elastic fiber staining of the blood vessels showed breast cancer vascular fiber disintegration, an uneven distribution, and stratification after HIFU irradiation. The results showed that HIFU damage increased with decreasing vessel diameter; smaller vascular diameter was positively correlated with more obvious damage by HIFU. HIFU can completely destroy the blood vessels that nourish breast cancers and damage larger blood vessels with elastic fibers. HIFU produces heat, which causes occlusion of the tumor’s vascular supply, leading to tumor cell death. This is important for preventing early breast tumor cell invasion, angiogenesis, and the formation of distant micrometastases.

UEAI staining vascular density count is commonly used to determine tumor angiogenesis status. UEAI staining was negative after HIFU treatment indicating that vascular endothelial cells that were in the process of hyperplasia had been completely destroyed (Fig. 6). VEGF expression level in HIFU group remarkably decreased than control group, which indicated tumor angiogenesis was significantly inhibited. Ultrastructural changes observed by electron microscopy further confirmed that HIFU treatment affects tumor microvessels.

Angiogenesis and tumor regeneration are interdependent and form a chaotic proliferation cycle. We conducted breast cancer tumor vascular HE staining, elasticity fiber staining, endothelial cell UEA staining vascular density count, observation of pathological changes by optical microscopy, and systematic observation of tumor vascular ultrastructural changes by electron microscopy. Our findings show that HIFU is a noninvasive treatment that can kill tumor cells and completely stop the proliferation of blood vessels; this may destroy the interdependence of tumor cell proliferation and blood vessel formation, thus disrupting the chaotic proliferation cycle. The necrotic tissue remaining after the tumor is killed is absorbed and gradually replaced by fibrous connective tissue [24].

Not like utilizing bevacizumab and other anti-angiogenic agents in breast cancer, breast cancer cells can escape anti-angiogenic therapy and develop resistance to anti-angiogenic agent, while HIFU ablation can make whole breast cancer tissue coagulative necrosis completely, so it cannot cause tumor resistance to HIFU ablation. The detailed mechanism needs further observation and exploration.

### Short-term follow-up after HIFU ablation

In our clinical observation, no severe complications were found in 25 patients after HIFU treatment, edema in HIFU group 25 patients appeared in the mammary tissue circumambient the ablated tumor. The edema gradually disappeared 7 days later. No skin burn and no infection, even hemorrhage occurred at the treatment site in all 25 patients.

The average follow-up time was 6–12 months in this study, the rate of local recurrence and distant metastasis was detected in both groups, and a local recurrence or a distant metastatic lesion was not detected in both groups. Because the follow-up period is short, the overall survival of HIFU treatment investigation in this study will be continued.

In a report by Wu et al., a safety margin of 1.5–2 cm around the ultrasound visible tumor was chosen to ensure complete tumor ablation, ultrasound-guided HIFU as adjunct to chemotherapy, radiotherapy, and tamoxifen for treatment of 22 patients with 23 malignant breast tumors (stages I to IV). Post-procedural biopsy revealed coagulation necrosis of the entire target tumor in all cases (100 %). After a median follow-up of 55 months, one patient died (5 %) and two patients (9 %) developed local recurrence. The 5-year disease-free survival was 95 % [24].

### Future prospects for HIFU ablation

HIFU ablation can make breast cancer and their vascularities coagulative necrosis thoroughly. It is demonstrated that HIFU treatment is an ideal localized tumor treatment that can irreversibly stop tumor growth and the resulting hyperplasia vascular necrosis while not damaging the surrounding normal tissues.

The benefits of HIFU therapy for breast cancer include the following: no bleeding, preserving the structure and function of breast tissue, no scarring, and little change to breast shape. Breast cancer surgery often requires complete hemostasis to avoid complications. When considering the merits of HIFU therapy, the prevention of bleeding-related complications is important. HIFU therapy is also highly repeatable and does not have radiation [9, 24].

As a noninvasive method, HIFU has a potential application in the treatment of patients with breast cancer. But clinical success of HIFU ablation completely relies on imaging technical monitoring and guidance. To use HIFU effectively, the ultrasound treatment device integrates with imaging system, either by using ultrasonography or MRI [9, 20, 24].

Our above clinical study proved that effective and safe HIFU therapy of breast cancer could also be obtained using ultrasonography guidance. High-frequency diagnostic ultrasound is sensitive enough to detect exact breast cancer margins, which aids in the complete destruction of breast tumors.

Comparatively speaking, ultrasonography is economical and flexible and can be performed in real-time and extensive availability whereas MRI can get high-resolution images and offer temperature measurement data. Image fusion may be the next important modality for real-time and effective guidance in breast cancers treated with the HIFU [9, 21].

#### Selection of suitable patients for HIFU ablation

One of the important subjects for successful HIFU ablation is suitable patient selection. Some breast cancer patients cannot be arranged for HIFU ablation for technical reasons.

This study only included women with breast cancer TNM staging (T1-2, N0-2, M0), single palpable tumors less than 5 cm in diameter, not included patients with nonpalpable lesions, multifocal breast cancer, and tumors close to the nipple, because it may face potential difficult for HIFU to target these types of breast tumors. For HIFU treatment, a distance of at least 1 cm between the tumor and the skin (to avoid skin burn) and tumor and chest wall (to prevent heat accumulation in the underlying ribs and lung) is required [24].

By strictly screening inclusion criteria, the patients for HIFU ablation only included small part of breast cancer cases in our above clinical study. It was shown that HIFU ablation can make breast cancer and their vascularities coagulative necrosis thoroughly.

In the past decades, screening programs and development in breast imaging techniques have led to an increase in the detection of early-stage breast cancer. Breast-conserving surgery with radiotherapy has become the gold standard treatment for localized breast cancer. To be equivalent to surgical removal, the effect of HIFU ablation should be to achieve total (100 %) tumor necrosis. Results of studies on HIFU breast cancer ablation to date have been variable, with histopathologic analysis demonstrating complete tumor necrosis in 20 to 100 % of patients treated [25].

Then, what can HIFU therapy do with nonpalpable and multifocal breast cancer (including axillary lymph nodes)? What is implications of local therapy for breast cancer and how to define the ablation margin for HIFU therapy?

Some uncertainties exist using HIFU ablation to treat breast cancer; thus, important indications can be gained from previous studies of conservative breast therapies involving surgery and radiation. Here, we give a comparison and review about breast-conserving surgery and HIFU ablation.

#### Multifocal breast cancer

Multifocal breast cancer is defined as the presence of two or more cancerous foci around the main malignant mass within one or multiple quadrants of the same breast, respectively. Invasive multifocal breast cancers are an important problem for ablation therapy because it is difficult to identify and destroy these clinically and radiologically negative lesions during HIFU ablation [9].

At the multivariate analysis, patients with multifocal tumors, once uniformly thought to be associated with a higher risk of local recurrence, and therefore treated with mastectomy, are now often offered breast conservation, when technically feasible, as most studies seem to indicate that the local recurrence rate is not higher in these cases than previous reports for unifocal cancers [26].

There are no published reports whether imaging-guided HIFU ablation can complete procedure of multifocal breast cancer.

Because of its noninvasiveness, pathologically negative margins cannot easily be ensured after HIFU ablation, and the margin status often must be assessed by imaging. Negative margins seen with imaging do not always represent pathologically negative margins.

For these reasons, it may not simply depend on imaging to confirm breast cancer before operation and assess treated effect after operation; it must be confirmed and followed up by puncture pathology, core-needle biopsy, if extracorporeal HIFU can be recommended as a conserving breast modality in the treatment of multifocal breast tumors.

#### Nonpalpable lesions and localization techniques

Breast cancer screening has dramatically increased the diagnosis of suspicious, nonpalpable breast lesions, and therefore also the need to localize them in order to plan surgical treatment. Furthermore, patients with a breast cancer removed with clear margins at the first excision seem to have a decreased risk of local recurrence compared with patients who need further re-excisions to achieve negative margins [27].

This represents a “hot” topic in breast surgery, since about half of breast cancers in modern surgical practices are nonpalpable, and this incidence is certainly destined to increase [25, 27].

Today, preoperative confirmation of malignancy is almost always achieved by puncture pathology, and therefore, we need to localize these small cancers to allow a one-step precise and directed excision [25, 27].

The wire localisation is still the gold standard. The wire localisation procedure is technically demanding and depends on both the wire placement by the radiologist and on the experience and three-dimensional orientation of the surgeon. The insertion of the wire can be uncomfortable for the patient; furthermore, there is a risk of wire migration between the time of insertion and the beginning of the surgery [28].

For this reasons several new techniques have been introduced in order to achieve breast tumor localization. Radio-guided occult lesion localization is a useful method to detect nonpalpable lesions through the injection of a nuclear tracer directly around the tumor under ultrasound or stereotaxic guidance. Then, the excision of the primary tumor is guided by a gamma probe, and a sentinel node biopsy can be performed at the same time if needed [25, 28].

Unlike the wire localization, the procedure is usually simpler and well tolerated, and the success rate is reported to be very high [25, 28].

A drawback is that two above guidance tools are invasive. Another technique for localization of nonpalpable breast tumors is represented by intraoperative ultrasound.

It satisfies most requirements for an ideal technique to localize nonpalpable breast tumors which are well visualized by ultrasound, while directing planes of surgery during the excision. This in turn is helpful in guaranteeing both negative margins and an adequate outline of resection in order to minimize the volume of excision. Identification rate of nonpalpable lesions and free margins of resection obtained through this procedure are extremely high [25, 29].

Retrospective study showed that ultrasonography-guided surgery for nonpalpable invasive breast cancer was more accurate than wire localization and radio-guided occult lesion localization because it optimized the surgeon’s ability to get proper margins (3.7, 21.3, and 25 % tumor-involved margins, respectively (*p* < 0.05). Intra-operative ultrasonography for nonpalpable tumors results in a remarkably lower rate of tumor-involved resection margins than palpation-guided surgery for palpable tumors (3.7 and 22.5 %, respectively (*p* < 0.05) [28].

Our above study was only including women with breast cancer of single palpable tumors, not including nonpalpable lesions. In this study, real-time ultrasonography was used to monitor the HIFU ablation course just like ultrasonography-guided surgery. These imaging techniques have been used for targeting the lesion to be treated, monitoring the therapeutic procedure, assessing the thermal effects on target tissue, and controlling the ultrasonic energy.

During the HIFU procedure, obvious changes in gray scale within the target tissue were immediately detected on the ultrasound image after HIFU ablation. The studies indicated that these hyperechoic zones corresponded well to the extent of coagulative necrosis and that there was a close relationship between the extent of necrosis as measured by gross examination and the hyperechoic extent measured on the ultrasonography immediately after HIFU ablation in both in vivo and in vitro tissues. In the clinical treatment of patients with breast cancer, it also found that the hyperechogenic regions were regular in shape and size and conformed to the extent of coagulative necrosis induced by HIFU ablation. Real-time diagnostic ultrasound guidance is seen to be useful in the detection of the coagulative necrosis of target tissue during HIFU procedures (9, 20, 24).

So from literature review and our above clinical observation, we believe that nonpalpable breast tumors may be treated with ultrasonography-guided extracorporeal HIFU as similar as palpable breast tumors treated in this study, if sufficient clinical trials are performed to confirm feasible for this modality at next step.

#### Methods for margin assessment and residual tumor detection after HIFU ablation

It is important to know the proper ablation margin because it is related to local recurrence and long-term survival. The amount of healthy breast tissue that should be destroyed and how to increase the probability of complete tumor necrosis in HIFU procedures are two issues under investigation. Studies of breast-conserving surgery can provide important information.

As conservative methods have developed in the last three decades and represent the standard of care for breast cancer patients around the world, the incidence of local recurrence has been widely studied. It occurs in 5–10 % of patients at 10 years, and it is more obvious in the first 3 or 4 years after primary surgery [25, 27].

Although several factors have been associated with the risk of local recurrence, at the multivariate analysis only age, status of surgical margins and postoperative radiotherapy seem to be independently correlated with it [25]. The result of patients with multifocal tumors offered breast conservation [26]. Similarly, infiltrating lobular carcinoma is probably not associated with a higher incidence of local recurrence compared to the ductal counterpart if resected with negative margins [25].

In breast cancer conservative treatment, there is no general agreement on the definition of “negative” margins and many describe such as the absence of tumor at the microscopic or inked margin, or with 1–3-mm clearance. It is clear that a high percentage of patients whose tumors are 2–5 mm from the radial margins have residual disease at re-excision. For this reason, and despite best efforts, as many as 20–25 % of patients in many institutions around the world return to the operating room after initial surgery for re-excision. While many reports fail to describe a statistically significant impact of margins on local recurrence, most would agree that one of the primary goals of conservative surgery is the removal of the primary tumor with a portion of normal breast tissue, so as to maintain a good breast shape [25–27].

The Dutch national guidelines for breast cancer stated, as a practical and reasonable guideline during surgery, the aim is to achieve a safe and cosmetically acceptable resection margin of 5–10 mm. The size of the tumor-free resection margin (>10 mm) is unrelated to local recurrence or overall survival. Higher risks of local recurrence have been shown only with evident involvement of the tumor on the inked resection margins. Therefore, there is no need to excise a tumor with a large volume of adjacent breast tissue. Accurate excision leads to a smaller and more precise volume of surrounding breast tissue removal without affecting the minimal tumor-free margin [28, 29].

Although the results of six prospective randomized trials in patients with invasive breast cancer have demonstrated that lumpectomy/quadrantectomy plus radiotherapy and mastectomy have equivalent survival results, it is worthwhile to remember that the first conservation trial, the Guy’s wide excision study initiated in the 1960s, has shown a decreased survival in the group treated conservatively. This suggests that poor surgical removal of the primary tumor, possibly with dubious margins and without inking of the specimen, together with the use of suboptimal postoperative radiotherapy, may lead to a negative impact not only on local control but also on survival [25, 30].

The meta-analysis showed that adjuvant radiotherapy after breast conservation surgery not only may improve local control but may also reduce 15-year breast cancer mortality. The effect of radiation on local control seems more obvious in node-positive patients, while the effect on survival remains important both for node-negative and node-positive patients [25–30].

Therefore, to complete surgical removal of the primary tumor with negative margins, surgical resection of breast cancer is performed with guidance by intra-operative palpation, the use of intra-operative ultrasonography, inking of the specimen to confirm resection margins. To prevent local recurrence and increase survival rate, radiotherapy is employed after surgical operation.

As a noninvasive method, clinical success of HIFU ablation breast cancer relies on imaging technical monitoring and guidance [9, 21].

By contrast to surgical therapy, we cannot completely depend on ultrasound imaging or MRI to determine the tumor margins, because the pathological diagnosis is the final diagnosis, while imaging of MRI, or ultrasonography is only as a method of adjutant diagnosis. For these reasons, it may not just simply rely on imaging guide to confirm negative margins of breast cancer before operation and to detect residual tumor after HIFU ablation; it must be confirmed and followed up by puncture pathology; and if puncture pathology proved with dubious margins, HIFU therapy can also be repeated and one advantage of HIFU therapy is that it has not radiation.

Based on breast-conserving therapy studies, a margin of at least 10-mm healthy breast tissue should be ablated to increase the probability of complete tumor necrosis [26]. Therefore, in our clinical study, image-detected breast cancer and 2.0-cm normal tissue surrounding the cancer were completely ablated to allow for the uncertainty that frequently exists concerning the exact location of definite tumor margins. However, for some superficial lesions close to the skin, there is a possibility of causing skin burns or leaving residual viable tumor cells if the overlying skin is left undamaged.

If HIFU is to be applied in clinical breast cancer treatment as a replacement to conventional surgery in the future, a reliable method for tumor margin assessment and detection of residual tumor after HIFU ablation must be available. Since HIFU treatment would lack a surgically excised pathological specimen, the tumor margin status could not be assessed by histopathological analysis. To date, most protocols have been treat-and-resect protocols, i.e., HIFU ablation followed by surgery, which allows histopathological tissue examination to evaluate the therapeutic effect of HIFU. Our above clinical study was adopted this modality.

In the few HIFU ablation studies where treated tumor tissue was left in situ after ablation, different strategies have been used to assess outcome. Furusawa et al. used clinical follow-up by MRI and ultrasonography every 3 months [31]. If one of the modalities showed suspicious findings suggestive for residual tumor, puncture pathology of the lesion was performed, and if residual tumor or tumor recurrence was found, the affected area was treated again with HIFU ablation. Gianfelice et al. used MRI follow-up at 10 days, 1 month, 3 months, and 6 months posttreatment to detect residual disease after HIFU ablation in high-risk surgical patients [32]. Additional to imaging, after 6 months, multiple puncture pathologies were performed through different areas of the ablation zone. In case of residual tumor, a second HIFU procedure was performed and followed by another targeted puncture pathology 1 month later. The technique of puncture pathologies for residual tumor detection along the ablation zone margins was also used by Wu et al. They performed puncture pathology at 2 weeks, 3 months, 6 months, and 12 months posttreatment [33]. In case of residual tumor, patients were subsequently treated with modified radical mastectomy.

Although no definitive guidelines are available today, the combination of ultrasonography (or contrast-enhanced breast MRI) and multiple puncture pathologies for residual tumor detection appears to be the most promising strategy for candidates after HIFU treatment where the treated tumor is left in situ.

#### Sentinel lymph node biopsy and management of special circumstances

SLN involvement is the single most important prognostic factor for survival in breast cancer patients, and consequently, information about it provide both staging information and guidance regarding treatment options [34].

SLN biopsy is now considered an adequate axillary staging procedure for patients who have breast cancer because it is easy to perform by experienced clinicians with less morbidity than axillary node dissection [34].

For the sentinel node procedure, a triple method will be used, consisting of combined lymphoscintigraphy (Tc99m colloidal albumin [Nanocoll®]), Patent Blue V® (Guerbet, Aulnay-Sous Bois, France) dye injection, and gamma probe detection; the sentinel node is sent for frozen section study. Node-positive patients who are preoperatively confirmed by ultrasound-guided cytological puncture will undergo an axillary lymph node dissection [28, 29].

Many concerns were raised in the past because SLN biopsy can result in some false-negative cases. A recent meta-analysis of 69 trials found the rate of false negatives to be about 7 % of the node-positive patients [25].

We performed breast puncture and SLN biopsy at the same time before treatment first, and then, it was proved by postoperative pathology. One false-negative case was found by axillary lymph node dissection pathology in control group (4 %), two false-negative cases were found by postoperative pathology in HIFU group (8 %), so SLN biopsy false negative rate was about 4–8 % of the axillary node-positive patients in our study. But no tumor vascular metastases status or SLN metastases status were observed in our study.

At the present time, surgical management and systemic options in case of SLN micrometastases are controversial. Most retrospective studies have reported a substantial rate of additional lymph node metastases in patients with SLN micrometastases, with a wide range between reports, making one think that patient selection is a key in determining the choice of candidates for completion lymph node dissection [25, 34].

More randomized trials will help to fully understand whether further axillary treatment should be mandatory when the SLN is positive [25, 34].

There are no published reports that describe imaging-guided HIFU ablation can complete procedure of axillary lymph node dissection now, because axillary lymph nodes with characteristic just like multifocal breast cancer, HIFU ablation of axillary lymph nodes may face some difficulty. These may depend on imaging-guided technical progress and the improvement of clinical practice in HIFU ablation.

But SLN biopsy acts as a role of patients’ arrangement, node-positive patients who are preoperatively confirmed by cytological puncture will accept an axillary lymph node dissection first, and then, imaging-guided HIFU ablation of breast cancer will be performed for them. As for methods for axillary lymph node dissection, we may choose endoscopy-assisted breast-conserving surgery or surgery operation directly currently.

Endoscopy-assisted breast-conserving surgery has the advantage of being less noticeable and having a small operative scar, whereas the indication of this method should be limited because of the difficulty in repair of a mammary gland defect via a small incision [35].

Tao Lu et al. [36] reported that early-stage breast cancer was treated by endoscopy carrying out axillary lymph node dissection, while ultrasound-guided HIFU ablated the breast cancer noninvasive at depth through the intact skin. Axillary lymph node dissection under endoscopy may be as another treatment approach without HIFU ablation of axillary lymph node available now.

#### A brief summary

This study only included women with early-stage breast cancer single palpable tumors. But as mentioned in the above literature, about half of breast cancers are nonpalpable tumors, which is not included multicentric lesions. If HIFU therapy is to be applied in clinical breast cancer treatment as a replacement to conventional surgery extensively in the future, a reliable method for tumor margin assessment and detection of residual tumor after HIFU ablation must be available.

Retrospective study showed that ultrasonography-guided surgery for nonpalpable invasive breast cancer was more accurate than wire localization and radio-guided occult lesion localization because it optimized the surgeon’s ability to get proper margins. Ultrasonography satisfies most requirements for an ideal technique to nonpalpable lesions and free margins of resection in surgical operation. In this above study, real-time ultrasonography was used to monitor the HIFU ablation course just like ultrasonography-guided surgery. Real-time diagnostic ultrasound guidance is seen to be useful in the detection of the coagulative necrosis of target tissue during HIFU procedures. We may adopt strategy of the combination of ultrasonography (or contrast-enhanced breast MRI) and multiple puncture pathologies for residual tumor detection; if residual tumor or tumor recurrence was found, the affected area was treated again with HIFU ablation.

We may still adopt treat-and-resect methods in nonpalpable and multicentric breast cancer, namely, HIFU ablation followed by surgery, which allows histopathological tissue examination to evaluate the therapeutic effect of HIFU.

With increasing cases of treat-and-resect modality and experience accumulation of HIFU ablation, HIFU ablation-treated breast tumor tissue may be left in situ after ablation, which is equivalent to surgical removal.

## Conclusions

We performed random clinical trials and adopted the modality of treat-and-resect protocols to evaluate the therapeutic effect of HIFU on breast cancer and their vascularities.

It was shown that HIFU ablation can make breast cancer and their vascularities coagulative necrosis thoroughly. Tumor angiogenesis and tumor regeneration are interdependent and form a vicious cycle that results in infinite proliferation. HIFU ablation can destroy all proliferating tumor cells and their feeding blood vessels at the same time; this may break interdependent vicious cycle of tumor angiogenesis and tumor growth. Not like utilizing bevacizumab and other anti-angiogenic agents in breast cancer, breast cancer cells can escape anti-angiogenic therapy and develop resistance to anti-angiogenic agent, while HIFU ablation can make whole breast cancer tissue coagulative necrosis completely, so it cannot cause tumor resistance to HIFU ablation. It may be a new anti-angiogenic strategy that needs further clinical observation and exploration. This study only included women with early-stage breast cancer single palpable tumors, not included nonpalpable and multifocal breast cancer, but the treatment indications may extend to the above type of breast cancer ultimately with technical progress and the improvement of clinical practice in HIFU ablation.

## References

[1] Chuan C, Yu-Bei H, Xue-Ou L, Ying G, Hong-Ji D, Feng-Ju S, Wei-Qin L, Jing W, Ye Y, Pei-Shan W, Yao-Gang W, Ke-Xin C (2014). Active and passive smoking with breast cancer risk for Chinese females: a systematic review and meta-analysis. Chin J Cancer.

[2] Punglia RS, Morrow M, Winer EP, Harris JR (2007). Local therapy and survival in breast cancer. NEngl JMed.

[3] Fisher B, Anderson S, Bryant J, Margolese RG, Deutsch M, Fisher ER, Jeong JH, Wolmark N (2002). Twenty year follow-up of a randomized trial comparing total mastectomy, lumpectomy, and lumpectomy plus irradiation for the treatment of invasive breast cancer. N Engl J Med.

[4] Nyström L, Andersson I, Bjurstam N, Frisell J, Nordenskjöld B, Rutqvist LE (2002). Long-term effects of mammography screening: updated overview of the Swedish randomized trials. Lancet.

[5] van Esser S, van den Bosch MA, van Diest PJ, Mali WP, Borel Rinkes IH, van Hillegersberg R (2007). Minimally invasive ablative therapies for invasive breast carcinomas: an overview of the current literature. World J Surg.

[6] Goldberg SN, Grassi CJ, Cardella JF, Charboneau JW, Dodd 3rd GD, Dupuy DE, Gervais D, Gillams AR, Kane RA, Lee FT Jr, Livraghi T, McGahan J, Phillips DA, Rhim H, Silverman SG, Society of Interventional Radiology Technology Assessment Committee, International Working Group on Image-Guided Tumor Ablation. Image-guided tumor ablation: standardization of terminology and reporting criteria. Radiology. 2005;235:728–39. 10.1148/radiol.2353042205 PMC3406173 15845798

[7] Van den Bosch MA, Daniel BL (2005). MR-guided interventions of the breast. Magn Reson Imaging Clin N Am.

[8] Tempany CM, Mcdannold NJ, Hynynen K, Jolesz FA (2011). Focused ultrasound surgery in oncology: overview and principles. Radiology.

[9] David S, Stanley B, Chris D, Wladyslaw G, Alexander K, James L (2013). MR-guided focused ultrasound surgery, present and future. Med Phys.

[10] Li S, Pei-Hong W (2013). Magnetic resonance image-guided versus ultrasound-guided high-intensity focused ultrasound in the treatment of breast cancer. Chin J Cancer.

[11] Schirner M, Menrad A, Stephens A, Frenzel T, Hauff P, Licha K (2004). Molecular imaging of tumor angiogenesis. Ann N Y Acad Sci.

[12] Staton CA, Brown NJ, Reed MW (2009). Current status and future prospects for anti-angiogenic therapies in cancer. Expert Opin Drug Discov.

[13] Bergers G, Hanahan D (2008). Modes of resistance to anti-angiogenic therapy. Nat Rev Cancer.

[14] Fakhrejahani E, Toi M (2014). Antiangiogenesis therapy for breast cancer: an update and perspectives from clinical trials. Jpn J Clin Oncol.

[15] Yadav L, Puri N, Rastogi V, Satpute P, Sharma V (2015). Tumor angiogenesis and angiogenic inhibitors: a review. J Clin Diagn Res.

[16] Giordano G, Febbraro A, Venditti M, Campidoglio S, Olivieri N, Raieta K, Parcesepe P, Imbriani GC, Remo A, Pancione M (2014). Targeting angiogenesis and tumor microenvironment in metastatic colorectal cancer: role of aflibercept. Gastroenterol Res Pract.

[17] Gong Z, Zhan R (1994). Pathological tissue production and dyeing technology, version 1.

[18] Vasudev NS, Reynolds AR (2014). Anti-angiogenic therapy for cancer: current progress, unresolved questions and future directions. Angiogenesis.

[19] Christina D, Mayer IA (2010). Antiangiogenic therapies in early-stage breast cancer. Clin Breast Cancer.

[20] Wang Z, Bai J, Li F, Du Y, Wen S, Hu K, Xu G, Ma P, Yin N, Chen W, Wu F, Feng R (2003). Study of a “biological focal region” of high-intensity focused ultrasound. Ultrasound Med Biol.

[21] Wu F, ter Haar G, Chen WR (2007). High-intensity focused ultrasound ablation of breast cancer. Expert Rev Anticancer Ther.

[22] Hynynen K, Chung AH, Colucci V, Jolesz FA (1996). Potential adverse effects of high-intensity focused ultrasound exposure on blood vessels in vivo. Ultrasound Med Biol.

[23] Yang R, Reilly CR, Rescorla FJ, Faught PR, Sanghvi NT, Fry FJ, Franklin TD, Lumeng L, Grosfeld JL (1991). High-intensity focused ultrasound in the treatment of experimental liver cancer. Arch Surg.

[24] Wu F, Wang Z, Chen W, Zou J, Bai J, Zhu H, Li K, Xie F, Jin C, Su H, Gao G (2004). Extracorporeal focused ultrasound surgery for treatment of human solid carcinomas: early chinese clinical experience. Ultrasound Med Biol.

[25] Alessandra M, Massimo F, Raffaella G, Vitelli CE, Lucio F (2010). Recent advances in the surgical care of breast cancer patients. World J Surg Oncol.

[26] Lim W, Park EH, Choi SL, Seo JY, Kim HJ, Chang MA, Ku BK, Son B, Ahn SH (2009). Breast conserving surgery for multifocal breast cancer. Ann Surg.

[27] Menes TS, Tartter PI, Bleiweiss I, Godbold JH, Seabrook A, Smith SR (2005). The consequence of multiple re-excisions to obtain clear lumpectomy margins in breast cancer patients. Ann Surg Oncol.

[28] Krekel NMA, Zonderhuis BM, Schreurs HWH, Lopes Cardozo AMF, Rijna H, van der Veen H, Muller S, Poortman P, de Widt L, de Roos WK, Bosch AM, van Amerongen AHMT, Bergers E, van der Linden MHM, de Lange de Klerk ESM, Winters HAH, Meijer S, van den Tol PMP. Ultrasound-guided breast-sparing surgery to improve cosmetic outcomes and quality of life. A prospective multicentre randomised controlled clinical trial comparing ultrasound-guided surgery to traditional palpation-guided surgery (COBALT trial). BMC Surg. 2011;11:8. 10.1186/1471-2482-11-8 PMC3069937 21410949

[29] Lloret Martí MT, Torregrosa Andrés A (2007). US-guided localization of nonpalpable breast cancer and sentinel node using 99 mTechnetiumalbumin colloid. Radiologia.

[30] Fentiman IS (2000). Long-term follow-up of the first breast conservation trial: Guy’ wide excision study. Breast.

[31] Furusawa H, Namba K, Nakahara H, Tanaka C, Yasuda Y, Hirabara E, Imahariyama M, Komaki K (2007). The evolving non-surgical ablation of breast cancer: MR guided focused ultrasound(MRgFUS). Breast Cancer.

[32] Gianfelice D, Khait A, Boulanger Y, Amara M, Beblidia A (2003). Feasibility of magnetic resonance imaging-guided focused ultrasound surgery as an adjunct to tamoxifen therapy in high-risk surgical patients with breast carcinoma. J Vasc Interv Radiol.

[33] Wu F, Wang Z, Zhu H, Chen W, Zou J, Bai J, Li K, Jin C, Xie F, Su H (2005). Extracorporeal high intensity focused ultrasound treatment for patients with breast cancer. Breast Cancer Res Treat.

[34] Wilke LG, McCall LM, Posther KE, Whitworth PW, Reintgen DS, Leitch AM, Gabram SG, Lucci A, Cox CE, Hunt KK, Herndon JE, Giuliano AE (2006). Surgical complications associated with sentinel lymph node biopsy: results from a prospective international cooperative group trial. Ann Surg Oncol.

[35] Ozaki S, Ohara M (2014). Endoscopy-assisted breast-conserving surgery for breast cancer patients. Gland Surg.

[36] Lu T, Bangyu L (2006). The progress of endoscopy combining with HIFU treatment early breast cancer. J Minimally Invasive Med.

